# Science-based health innovation in Rwanda: unlocking the potential of a late bloomer

**DOI:** 10.1186/1472-698X-10-S1-S3

**Published:** 2010-12-13

**Authors:** Kenneth Simiyu, Abdallah S Daar, Mike Hughes, Peter A Singer

**Affiliations:** 1 McLaughlin-Rotman Centre for Global Health, at the University Health Network and University of Toronto, MaRS Centre, South Tower, Suite 406, 101 College Street, Toronto, Ontario, M5G 1L7, Canada; 2 Ministry of Education, Rwanda

## Abstract

**Background:**

This paper describes and analyses Rwanda’s science-based health product ‘innovation system’, highlighting examples of indigenous innovation and good practice. We use an innovation systems framework, which takes into account the wide variety of stakeholders and knowledge flows contributing to the innovation process. The study takes into account the destruction of the country’s scientific infrastructure and human capital that occurred during the 1994 genocide, and describes government policy, research institutes and universities, the private sector, and NGOs that are involved in health product innovation in Rwanda.

**Methods:**

Case study research methodology was used. Data were collected through reviews of academic literature and policy documents and through open-ended, face-to-face interviews with 38 people from across the science-based health innovation system. Data was collected over two visits to Rwanda between November – December 2007 and in May 2008. A workshop was held in Kigali on May 23rd and May 24th 2009 to validate the findings. A business plan was then developed to operationalize the findings.

**Results and discussion:**

The results of the study show that Rwanda has strong government will to support health innovation both through its political leadership and through government policy documents. However, it has a very weak scientific base as most of its scientific infrastructure as well as human capital were destroyed during the 1994 genocide. The regulatory agency is weak and its nascent private sector is ill-equipped to drive health innovation. In addition, there are no linkages between the various actors in the country’s health innovation system i.e between research institutions, universities, the private sector, and government bureaucrats.

**Conclusions:**

Despite the fact that the 1994 genocide destroyed most of the scientific infrastructure and human capital, the country has made remarkable progress towards developing its health innovation system, mainly due to political goodwill. The areas of greatest potential for Rwanda are in traditional plant technologies. However, there is need for investments in domestic skill development as well as infrastructure that will enhance innovation. Of foremost importance is the establishment of a platform to link the various actors in the health innovation system.

## Background

In the years between 1996 and 2005, Rwanda registered an impressive annual economic growth rate averaging about 8 per cent [1]. This growth took place despite the fact that Rwanda is one of Sub-Saharan Africa’s smallest countries, and had just emerged from a traumatic history of political upheaval that culminated in genocide in 1994. The country’s population of 9.5 million [2] inhabits a landscape of only 26,000 sq km, making it one of Africa’s most densely populated countries. Its domestic economy is characterized by low technology enterprises dominated by commodity trading and basic services. The country is faced with many structural challenges—it is land-locked, has very few natural resources, and relies almost entirely on earnings from exports of high volume and low value agricultural products such as tea and coffee, making it very susceptible to the whims of the global market.

Rwanda’s economic growth, so soon after the destruction of the country’s infrastructure and human capital during the genocide, was a result of several factors including strong political leadership, much international support, and the zeal and optimism of its people. Although the economic growth rate dropped to 5 % in 2008 [3], mainly due to bad climatic conditions that affected its agricultural exports, it still compares favourably to most countries in Sub-Saharan Africa.

Rwanda’s impressive economic growth has not, however, yet been fully translated to improved socioeconomic status for all segments of the population. The country’s per capita GDP at only US $230 (41% from agriculture, 38% from tourism and 21% from industry, mainly construction) means that poverty levels are still high [4], with more than 64 % of all households in the country living below the poverty line. 85 % of those living below the poverty line are in rural areas. Only 41% of the population had access to safe drinking water in 2008, while just over 50 % had access to safe sanitation [5]. As a result, the incidence of diarrheal diseases is high, partly contributing to the high infant mortality rate which, at 119 per 1000, remains well above sub-Saharan Africa’s average [6]. Such factors coupled with the high incidence of malaria and HIV/AIDS limit the average life expectancy to only 49 years.

Rwanda’s national innovation system may be described as still very underdeveloped, but changes that have occurred in the country’s economy since the genocide are positively reflected in the innovation system. The country still ranks very low in knowledge indicators, having an innovation index of 1.22 out of 10 [7], having less than 30 ISI publications between 2002 and 2004 [8] and zero patents between 2000 and 2004 [9]. However it has made tremendous investments to improve this, including, for example, providing universal free primary school education, expanding higher education opportunities, and establishing collaborations and training programs with foreign universities. In fact, the number of students in higher education has risen from only around 1,000 students in one University in 1994 to more than 27, 787 in 2005, 39% of them female [10]. The domestic scientific skill base remains weak, but the government is attempting to counter this by pursuing a very liberal policy towards hiring of appropriate expatriate staff, e.g., nationals of the East African community member countries do not require work permits to work in Rwanda. The government is also encouraging members of the Rwandese diaspora to lend their expertise. To bolster the nascent private sector and the almost non-existent manufacturing base, reforms have been introduced to ease the cost of doing business in Rwanda. These include hastening the pace of registration of new businesses as well as increasing efficiency in enforcing contracts [11].

In this paper we present research on science-based health innovation in Rwanda, including biotechnology. By science-based health innovation, we mean technological innovation across a spectrum of sophistication, from vaccines, pharmaceuticals, and medical devices to some plant medicines where attempts to scientifically standardize or characterize medicines have been made. We take a broad definition of innovation as not only new-to-the-world innovation, but also the diffusion, adaptation and use of technologies. We use the OECD definition of biotechnology: ‘the application of science and technology to living organisms, as well as the parts, products and models thereof, to alter living or non-living materials for the production of knowledge, goods and services’ [12].

The purpose of this paper is to describe and analyze Rwanda’s science-based health innovation using an innovation systems framework, which takes into account the wide variety of stakeholders who contribute to the innovation process and emphasizes the dynamic interaction and knowledge flow between them [13]. To date there has been little research on science-based health product innovation in Rwanda. As far as the authors are aware, this is the first study to explicitly look at this topic in Rwanda.

The study was undertaken at the invitation of the former Minister in the Office of the President in charge of Science and Technology. The 1994 genocide marked a watershed moment for the country in general and the national innovation system in particular. Most of the already modest research base and private sector was destroyed. Our evaluation of the innovation system has this background context in looking at developments in the post-genocide period of the last 15 years.

## Methods

A case study research methodology was used in this study [14]. Data were collected through reviews of academic literature and policy documents and through open-ended, face-to-face interviews in Rwanda. Interviewees were identified through purposive and snowball sampling; we interviewed 38 people from across the science-based health innovation system, including government officials (n=8), researchers in research institutes and educational institutions (n=11), entrepreneurs (n=6), international donors (n=2), and non-governmental organization representatives (n=5), over two visits to Rwanda between November – December 2007 and in May 2008. Given the important role of the members of the diaspora, we also interviewed 6 members from them. Following the interviews, a workshop was held in Kigali on May 23^rd^ and May 24^th^ 2009 involving the interviewees and other stakeholders to validate the findings. A business plan was then developed to operationalize the findings. We report the findings of our case study analysis below.

All quotes are from the interviews unless noted, and with permission. This study was approved by the Office of Research Ethics of the University of Toronto.

## Results and discussion

In the following sections, we describe and discuss Rwanda’s science-based health innovation system.

### Government

The integration of science and technology into the country’s economic development has been given prominence in Rwanda since 1994. President Kagame has emphasized “the imperative of focusing and utilizing science and technology,” to effect the country’s and Africa’s socioeconomic transformation. In a speech to the 8^th^ summit of the heads of state of the African Union, President Kagame reiterated the importance of innovation, saying *“It is about applying science and technology holistically - in all levels of education and training, in ‘commercializing ideas’, developing business and quickening the pace of wealth-creation and employment-generation, in enabling government to provide better services, and indeed in providing basic tools to society at large for self- and collective betterment.”*[15].

The guiding document for recent growth in science and technology in Rwanda is the 2006 National Science, Technology, Scientific Research and Innovation policy [16]. Included in the policy are plans “to apply science to Rwanda's problems in health, agriculture and the environment” by increasing crop yields and improving animal husbandry through the use of biotechnology. In addition, the policy paper envisions setting up district-level 'innovation centers' and national technology parks to encourage research and development, particularly by small businesses.

Science and technology activities in Rwanda were transferred to the Ministry of Education in 2009. Prior to that, the Ministry in the Office of the President in charge of Science and Technology was the central agency in Rwanda’s science and technology model, and directed both funding and research orientation. However, it was understaffed, with only four staff who had to achieve the wide goals of the ministry as well as coordinate all science and technology research and all aspects of information communications technology in Rwanda.

The Ministry’s 2009/2010 budget shows enhanced commitment to innovation, with line items included for establishing linkages between academia and industry and for strengthening intellectual property.

Though the country lacks a biotechnology policy, this remains one of the priority areas as alluded to by the then Minister of Science and Technology Prof. Romain Murenzi who when interviewed said, *“I was thinking of the need to establish a biotechnology authority that will integrate biotechnology in health and agriculture”*. However, a national bio-safety framework [17] was drafted in 2005, mainly to protect human health and the environment due to applications of biotechnology.

Rwanda’s public health goals are spelled out in the “Health Sector Policy-Government of Rwanda” document published in 2005 [18] among which is the improvement of the availability of quality drugs, vaccines, and consumables and a goal to strengthen national referral hospitals and research and treatment institutions through collaboration with other ministries and agencies. Within this document most of the research focus is limited to clinical aspects of health as opposed to health innovation.

As a “Least Developed Country”, Rwanda in 2008 became the first country to take advantage of the World Trade Organization (WTO) amendment to the TRIPS (Trade Related aspects of Intellectual Property) Agreement [19], when it awarded a tender to the Canadian generic manufacturer Apotex to manufacture the triple combination AIDS drug "Apo Triavir" on its behalf. The first shipment of 6.8 million pills arrived in Rwanda in September 2008. The objective is to increase accessibility to cheap anti-retroviral (ARV) drugs to people with AIDS in Rwanda, many of whom are poor. Government officials stated that they hope by 2016, when the TRIPS agreements take effect, Rwanda will have developed capacity to manufacture its own pharmaceuticals.

An effective intellectual property regime is needed to support innovation in Rwanda. Despite an intellectual property law being passed by parliament in 2008, information has yet to reach scientists, and this law has yet to fully take effect. As put by Mark Bagabe, former Director-General of the National Agricultural Research Institute (ISAR), “*We really have not internalized the need to patent our research. We are still rebuilding and focused on basic research and publication. In fact, as an institute, we have lost so much patentable information because of this*”. There have been several missed opportunities where Rwanda could have benefited, but because there are no proper policies on innovation management, the country has not benefited. For example, a fertilizer was formulated at the Institut de Recherche Scientifique et Technologique (IRST) by a scientist who refused to disclose his formula; when he moved from the institution, he went away with the knowledge. Bean seed varieties developed at ISAR have also been found being marketed in countries such as Malawi, yet the Institute does not receive any royalties. And sericulture lines developed by ISAR are not shared with the textile industry in Rwanda.

Rwanda is a member of the WIPO convention, the constituent instrument of the World Intellectual Property organization. However, there are no IP attorneys in Rwanda and respondents in this study doubted whether the country’s judiciary was sufficiently equipped to handle IP disputes. Another stated limitation which is common to many African countries was the high cost to file, maintain, and enforce patents, all which are the responsibility of the inventor; hence if the invention has no immediate financial returns, there is reduced willingness to attempt to patent.

Rwanda’s drug and medical product regulatory capacity is also very limited. The Food and Drug authority has just been established but has yet to become fully operational. Limited staffing and infrastructure issues, as well as the porous nature of Rwanda’s borders, mean that it is difficult to enforce drug quality and drug registration. Counterfeit products have been found in pharmacies in Rwanda [20] and this is not only a drain on the economy, because patients are wasting money, but also a significant health risk.

### Research institutes and universities

Like many African countries, the largest and most active R & D institutions in Rwanda are public sector ones involved in agricultural research. This is not surprising, as the economy is heavily agrarian. The main focus has been rural development and poverty alleviation, with little emphasis on basic and applied R & D. Up until 1994, political upheavals, changing budget priorities, staff purges due to ethnic wrangles, and generally poor morale left institutions incapable of carrying out their core mandates. Since then, investments in science and technology by the government and donors have improved the situation.

At the base of the educational system, 96% of primary-school age children now receive free education [10]. The percentage of students progressing from primary school to secondary school is still low, perhaps due to absence of universal secondary school education. But overall, there has been a very significant increase in the number of students graduating from institutions of higher learning in Rwanda over the last 15 years.

The number of students in higher learning institutions increased from 10,000 in 2002 to 27,787 in 2005 [21]. Rwanda has six public and several private higher education institutions [22]. Data from the 2003 census showed that there were then 0.5% of university graduates in the population, compared to the African average of 4%. However, the gross enrolment rate at tertiary level was 3.2%, which is regionally comparable. Until recently there was no graduate training at the National University of Rwanda, forcing many students to be sent abroad for training.

#### Institute of Scientific and Technological Research

The Institute of Scientific and Technological Research, also known as the Institut de Recherche Scientifique et Technologique (IRST), was established in 1989. It is the country’s premier medical research institute, including research in phyto-medicine, biodiversity, alternative energy sources, environmental studies, and other fields related to medicine. Its website indicates that there is a department of biotechnology, but a visit to the institution revealed that this has not yet been established. The Institute has acknowledged the importance of domestic innovation and has established an innovation and technology transfer unit, as well as organized seminars to sensitize its scientific staff on intellectual property management since 2005. According to the Institute’s Director General Dr. Jean Nduwayezu, in 2007 the institute had a total of 96 staff (88 involved in research) but only 6 PhDs (2 Rwandese nationals and 4 foreigners). He also stated that the optimal number of staff required is 121, hence there is a shortfall of manpower.

The Centre for Research in Phyto-medicines and Life Sciences at IRST carries out research in traditional medicines, by liaising with traditional healers and carrying out safety and anti-microbial efficacy tests. Material is sent to international laboratories, mainly in Europe, to carry out further investigation on plants that look promising. This has proved difficult as they first have to identify effective partners abroad. In some cases, results of material sent abroad are never communicated back to the scientists. Scientists expressed fear that they could be losing intellectual property this way. As one scientist remarked, “*Sometimes all it needs is for an experienced scientist with the necessary equipment to establish anti-microbial properties of a plant. They then go ahead and make derivatives from it that are active against different pathogens and patent it claiming it to be theirs”*.

Currently the main focus is testing for toxicity using mice on traditional plants submitted by the traditional healers. After ascertaining safety, the institute’s logo is usually affixed on the product which allows the traditional practitioner to market the product. Examples of traditional medicine extracts now being marketed include products for such mild common ailments as diarrhoea and common colds as well as ointments to relieve pain. An earlier survey of traditional medicinal products [23] showed examples of such medicines to include an anti-spasmodic syrup from *Datura stramonium* (Gifurina), anti-cough syrup from *Plantago lanceolata* (Bentakor), cough syrups from the mixture of *Eucalyptus globulus* and *Datura stramonium* (Tusinkor), and from *Eucalyptus globulus, Datura stramonium* and *Thymus vulgaris* (Tumitusilinga), a mouth disinfectant solution from *Eucalyptus globulus* and *Mentha sacchalinensis* (Kanwalina), an anti-arthritic formulation from *Capsicum frutescens,* an anti-inflammatory ointment from *Calendula officinalis* (Calendula) and an antiscabies ointment ‘Tembatembe A’ containing a rotenone compound from *Neorautenenia mitis*, which was used successfully on prisoners.

#### National Agricultural Research Institute

The National Agricultural Research Institute, also known as the Institut des Sciences Agronomiques du Rwanda (ISAR), is Rwanda’s major agricultural R&D agency. ISAR, the oldest scientific research institution in Rwanda, was rebuilt after the 1994 war; this included the construction of new laboratories and installation of modern scientific equipment. Many staff members have received international training (Masters and PhD) and today the institute can be described as the institution that has the best capabilities within Rwanda to carry out basic biotechnology research. The institute has the capacity to carry out limited molecular biology techniques, tissue culture, and food fortification. However, at the time of the study it had no clear IP policy and very little focus on innovation. Like most other research institutes in Rwanda, there had been very little linkages with the private sector.

In addition to the above two research institutes, higher education institutions in Rwanda are participating in research.

#### National University of Rwanda

The National University of Rwanda is the country’s biggest university and was established in 1963. Located at Butare, it offers a wide range of degree programs. It has also developed a new strategic plan in which great emphasis has been placed on scientific innovation. The Faculty of Agriculture and the School of Medicine are the departments that are actively involved in health related research. However, because of the high student to staff ratios at the university, resources are heavily skewed towards teaching at the expense of research. As one professor remarked, *“Our focus at the moment is to teach. Because we have very many students compared to the number of staff, we cannot conduct any research.”* Other limitations include lack of scientific infrastructure as well as general funding. Currently, most health research is limited to epidemiological studies and clinical research on behalf of international organizations and foreign university researchers.

#### Kigali Institute of Science and Technology

Kigali Institute of Science and Technology (KIST) was the first public technological institute of higher learning in Rwanda. Though originally established as a technology and engineering institution in 1997, it has expanded its curriculum to many other areas including health sciences and now offer degrees in various subject areas. In 2009, it established a faculty of medical biotechnology, to train both undergraduate and graduate students. As the Rector, Prof. Abraham Atta Ogwu said, *“We realized the future importance that medical biotechnology will play in trying to address Rwanda’s health needs, and that is why we decided to be the first institution in Rwanda to offer training specifically geared towards medical biotechnology”.* KIST has also established the Centre for Innovation and Technology Transfer to develop appropriate technology solutions for rural areas and has an incubation facility that nurtures young entrepreneurs, providing basic facilities and business management training services to them.

According to Ministry of science officials, KIST is more adaptable to the country’s needs because it does not have historical bureaucracy that other institutions that have been there for a long time face. In addition there are many international visiting scholars at the institution as well as international collaborative agreements with other institutions enabling knowledge exchange that is critical for developing skills in innovation.

Though it was difficult to obtain, for any of the country’s institutions, the allocation of research budgets across salaries and capital costs, there was clear evidence that the bulk of resources are currently allocated towards operating costs, thereby affecting the efficiency of R & D.

### Private sector

Rwanda’s private sector is nascent, with most businesses being small scale and with low technology enterprises. From the interviews many entrepreneurs were young and were western educated mainly in the United States and Britain and had only returned to the country in the late 1990s or early 2000`s.

Private sector investors have not generally been involved in exploiting any potential opportunities from research, let alone from research in health or biotechnology.

One firm that has tried to produce local health products is Ikirezi Natural Products, a firm that has been involved in extraction and export of geranium oil, which is used as an essential oil in remedies for dermatological conditions. According to the founder, this has generated significant economic benefits to farmers, as the value from the same acreage of land for geranium is much higher than for coffee and tea – demonstrating the potential of alternative crops.

Also interested in health technology is Rwanda’s largest industrial manufacturer, Utexrwa, which specializes in textile manufacture. The company CEO has expressed interest in manufacture of pyrethrum impregnated mosquito nets which would serve two purposes: provide a market for pyrethrum that is currently being exported unprocessed outside the country, and save the importation of long lasting mosquito nets. There are talks between the firm and universities in Canada about developing technology that could use pyrethrum to replace synthetic pyrethroids on Long lasting Insecticide-treated Mosquito nets.

One other area examined in this study was access to risk capital and credit. We identified at least four venture capital firms in the city of Kigali, plus several banking and micro-finance institutions. The Rwandan Enterprise Investment Company (REIC) is the largest such provider of risk capital. The company’s legal foundation, established by an act of Parliament, is viewed as a source of strength as it is a public institution with private sector goals. Its primary responsibility is to government, but it has autonomy in its investment decision making. The organization has the mandate of financing projects which have both commercial and social benefits. According to the CEO of REIC, the major limitation to investment in health R & D has been the inability for them to determine the commercial viability of health research in Rwanda. *“We have this mandate towards looking at key core areas that need financing because nobody else is willing to finance them”* says Mike Kagwa, its CEO. “*If health is an asset that can be structured, we will be in it. Not many people know how to structure health research as a viable asset and we don’t know where the commercial gain is, except social benefits”*.

Foreign venture investors, especially American firms, can also be found in Rwanda. While many are driven by their conscience to help rebuild the country, a number of them are simply looking for potential profits that can be reaped from a country that is starting from scratch, as there is very little domestic competition. *“My investors are hard-nosed capitalists”,* says American trained Antoine Bigirimana, CEO of Kigali based Thousand Hills Venture Capital. “*They are simply looking for ideas that are easy winners”*.

However, there is a lack of connection between capital providers and researchers in the university and research institutes. This is due to the absence of knowledge flow and of venues in which they can meet and exchange ideas. When asked, most researchers in Rwanda were unaware of the existence of local venture capitalists, while the venture capitalists were also unaware of the fact that there might be technologies stagnating on shelves in local institutions.

Private sector entrepreneurs in Rwanda are organized under the Rwandan Private Sector Foundation. We were informed by one of the interviewees that this umbrella organization of businesses in Rwanda has eight regional centres where it trains small business management skills. There are also a number of micro-finance institutions that mainly support small businesses by providing them with loans and business training.

The only pharmaceutical manufacturer in Rwanda is the government owned Pharmaceutical Laboratory of Rwanda (LABOPHAR). After years of loss making it was recommended for privatization. The company was initially set up to manufacture quinine and a variety of generic products, but has been limited to manufacturing intravenous fluids and water for injection, i.e. bulk products that cannot be imported and yet are necessary for the country’s hospitals. According to some interviewees, the poor performance of the company is mainly attributed to lack of technical know-how, lack of infrastructure, poor management, and the fact that it was a government-run institution without the drive of private entrepreneurship. It thus became a burden to the Rwandan tax payer, hence its privatization.

Drug distribution in Rwanda also appears ad-hoc, mainly because the drug regulatory agency has not been fully staffed. Individuals can import any products without any restrictions or assessment on their quality. As such, many pharmacies in Rwanda import directly, mainly from India and Kenya. CAMERWA is the government agency charged with drug procurement and distribution and mainly supplies government-run hospitals with drugs, all of which are funded by donor agencies. The agency is reasonably well staffed at the national office, but lacks the infrastructure needed to ensure successful national distribution.

Innovation is also affected by market factors. The domestic market in Rwanda is small, both in size and purchasing power, hence demand for products is limited. This tends to affect the quantity and type of products that are developed for the Rwandan market by domestic and international enterprises.

Overall, Rwanda’s general business climate is well-regarded in comparison to its neighbouring countries in the East African Community and in Africa in general. For example, Rwanda has less corruption and bureaucracy than the regional standards, and the time taken to start a business is shorter than the average for sub-Saharan Africa – all factors which make local science-based health innovation easier. Some business issues that Rwanda is currently addressing include legal and regulatory constraints as well as improvements to enforcement and employment practices.

### NGOs and donors

Non-governmental organizations and donors also influence Rwanda’s science-based health innovation. The Rwandan government receives more foreign aid per capita than most countries in Sub-Saharan Africa [24]. This aid is delivered through a mix of budget support and project financing. At its peak in 2005, 60% of the national budget was directly funded by donors. Many interviewees said that because scientific research in Rwanda is largely financed by donors and multilateral development banks, channelled through both the national government and project support directly to various institutions, the allocation of resources across various lines of research reflects donor influence. Most donors fund specific programs within their traditional areas of interest. Thus the European Union funded construction and equipment of a coffee research laboratory at ISAR to stimulate coffee research, reflecting their priority of agriculture.

The government has made efforts to gradually reduce this to just over 40 % in 2009. According to officials in the Ministry in charge of science, the main donors in science and technology are the World Bank, the African Development Bank, and the British international development agency, DFID. DFID is currently assisting in the development of an institutional framework within the Ministry of Science and Technology that will, among other things, support innovation platforms. The World Bank is working on programs that enhance scientific capacity, focusing on value addition of agricultural products. The aim of this World Bank project is to foster economic growth by creating jobs and wealth through building an economy that is driven by innovation and higher-value production systems.

In 2006/7, the African Development Bank, through the science and technology sector budget support, assisted in drafting the Science and Technology in Education work plan. This plan, which has been put in place, aims to foster the teaching of science and technology from the primary school level with the provision of science corners in all schools. At the secondary school level, plans include the building of science labs. Vocational and technical schools in Rwanda are also to be renovated and equipped to provide effective education geared to the labor market.

Ministry of Science officials also told us that collaborative programs with institutions in the US and other developed countries have also been established with the main purpose of providing training to Rwandese scientists. These include Carnegie Mellon and Massachusetts Institute of Technology (MIT) whereby students from Rwanda are given scholarships to train at these institutions in the fields of computer science and engineering. The Government of Rwanda has also established a Presidential Scholars program in collaboration with universities in the US that enables Rwanda’s best math and science students to receive four-year scholarships to do their undergraduate studies at US colleges and universities. The major institution supporting the Presidential Scholars program is Oklahoma Christian University. The focus of the studies are civil and electrical engineering, computer science, chemistry, and architecture, and biotechnology, areas of study deemed critical to Rwanda’s long-term, economic development plans.

The National Treatment and Research AIDS Center (TRAC) is a non-governmental organization that tries to shape health policy in Rwanda using treatment outcomes. TRAC partnered with the software company Voxiva to develop an integrated software system for monitoring diseases in Rwanda. Using both web-based software and mobile phones, TRACNET collects, stores, retrieves, displays and disseminates critical program information, as well as managing drug distribution and patient information related to the care and treatment of HIV/AIDS.

### Diaspora

The displacement of many of Rwanda’s citizens because of historical strife resulted in a large Rwandan diaspora. According to the Ministry of Foreign Affairs, an estimated 6 million people of Rwandese descent live abroad, mainly in Europe and North America; this number is over half the entire population of people living in Rwanda. This significant resource can act as a source of revenue through remittances and direct investments, as well as a pool from which to draw ideas, skills, attitudes and technologies. Discussions with Jean Kimanzi, President of Rwandan Diaspora, revealed that there are already two programs – Transfer of Knowledge Through Expatriate Nationals (TOKTEN) and MIDAS – that enable qualified Rwandese in the diaspora to travel back to Rwanda periodically to assist in transfer of knowledge. These programs aim to supply Rwanda with short-term expertise not readily and immediately available locally. “The environment is right. There is political will and Rwandese should take advantage of this to give something back to their country” remarks Dr. Kimanzi.

## Conclusions

### Strengths and good practices

Rwanda’s science-based health innovation system including biotechnology has several strengths, which include liberal policies on employment of expatriates, the clustering of its academic and research institutes in one main geographical area, and the multi-lingual nature of the population, which means that it is easier for Rwanda to trade with many countries.

Perhaps the greatest strength of Rwanda’s science and technology system is the political support and dynamic leadership that the country is currently experiencing, especially from the country’s President, Paul Kagame. This was cited by many respondents who also mentioned the fact that the country has made dramatic investments in science and technology. According to the President, Rwanda currently invests 1.6% of its GDP on Science and Technology [25]. The country has also developed a strong science, technology and innovation policy, meaning that Rwanda has realized the need to incorporate STI in its economic development agenda. Activities to implement the policy have already started and the World Bank carried out a needs assessment based on the STI policy and recommended practical solutions to some of Rwanda’s problems [26]. The main area of focus was value addition to agricultural products. The 2009 government budget provided funds (approximately 100, 000 dollars) to support innovation activities. These funds can be accessed by any scientist or entrepreneur. An initial disbursement of these funds was made to support projects in KIST and ISAR; one on the use of banana fibres in textiles and the other on sericulture development in Rwanda. From interviews with government officials, it also appears that the government readily welcomes donors and international institutions that are willing to help it strengthen science activities in Rwanda.

However, while Rwanda has a good STI policy, STI activities in the country are mainly focused on the development of information technology with little focus on health and health biotechnology. The reasoning behind this is that Rwanda aims to become the information technology hub of Africa. Information technology has become very useful in supporting health programs in Rwanda, as evidenced from the TRACNET program. However, interviewees mentioned that health innovation is not sufficiently mentioned in the STI policy.

The main opportunities in science-based health innovation and biotechnology for Rwanda are in traditional plant technologies (see Table 1 for selected opportunities). Interviewees repeatedly cited this as the area of greatest potential. Reasons included the existence of IRST (an institute that has some capacity to carry out research in traditional medicines), the ready availability of a variety of traditional plants, and the experience of traditional plant healers. Health-related information technology was also identified as an area with great potential. Many interviewees gave the reason for this to be the well developed information infrastructure in the country, as well as the government’s inclination to support information technology policies.

**Table 1:** Various products and processes being developed in Rwandan institutions

Product	General Area	Organization	Description
Traditional herbal medicines -Gifurina-*Datura stramonium* -Bentakor-*P. Lanceolata* -Tusinkor-*E.globulus* -Tumitusilinga-*T.Vulgaris* -Kanwalina-*M.Sacchalinensis* -Calendula-*C.officinalis* -Tembatembe A-*N.Mitis.*	Anti-spasmodic Anti-cough Anti-cough Oral disinfectant Anti-arthritic Anti-inflammatory Scabies	IRST	Whole plant extracts. Adoption of existing practices by traditional healers and carry out safety and efficacy tests

Essential oils-Geranium	Cosmetics / Dermatology	IKIREZI Natural products	Extraction of essential oils from geranium plant

Intravenous fluids and water for injection		Laborphor	For use in hospitals, since transport costs for this bulky product are high

Pyrethrum treated long lasting mosquito nets	Malaria	Utexrwa	Concept under development with researchers from Canada

Health information technology software	Health IT	TRAC	Developing software for integrating health information, in partnership with Voxiva.

### Recommendations

From the interviews, we can conclude that there are specific challenges that need to be overcome for Rwanda to successfully exploit its science-based health innovation and biotechnology. When we interviewed the management of the various research institutions, they cited deficiency in scientific infrastructure and a shortfall in the number of trained scientists who are capable of carrying out advanced health biotechnology research as one of the major challenges. Specifically, many interviewees cited the low number of people trained in molecular biology techniques as a hindrance to development of the sector. Post-graduate opportunities within Rwanda are limited, particularly within health biotechnology.

In addition, there is a skewed distribution of health researchers in favour of teaching at academic institutions. This has contributed to the dismally low number of research publications by local Rwandan scientists, which total only 30 research articles published between 2002 and 2004. From the interviews, many of the scientists did not appear able or sufficiently motivated to pursue entrepreneurial activities as most do not consider research to be rewarding.

The brain drain that occurred during earlier years in the country’s history continues to plague science and technology. This was made worse by the genocide, during which scientists were among those who were killed or fled to other countries in the region and abroad. While a few have returned and formed part of the interview pool, many are still abroad. However, this may also represent a strength, as the Rwandan diaspora consists of highly qualified individuals who left the country either as refugees or to pursue higher education in the West. When interviewed, many members of the diaspora said that since they are now occupying senior positions in new countries, they are unwilling to return to Rwanda, but they could lend their skills to support development of health biotechnology if appropriate policies are put in place.

Another problem is brain drain within the country, mainly as a result of a serious mismatch between capabilities, opportunities and compensation of scientists within Rwanda. Interviewees repeatedly cited the efflux of researchers from university and research institutes towards sectors that do not engage in research, such as NGOs that offer attractive work environments and higher salaries.

Discussions with scientists showed that a career in politics has become a very attractive field for Rwandan scientists. Indeed, a close examination of the Rwandan Cabinet and Parliament reveals a contingent of very highly trained professionals, many of whom possess advanced degrees in biotechnology, engineering, or health and significant research experience. This illustrates the internal brain drain phenomenon, although it may be argued that having a scientifically literate political establishment has contributed to increased support for science and technology.

We can deduce from the study that science-based health innovation and biotechnology in Rwanda is currently fragmented, with training and scientific research falling under many different agencies. For example, universities fall under the Ministry of Education, while research institutes fall under different ministries including the Ministry of Agriculture (e.g ISAR, ISAE) and the Minister in President's Office in Charge of Science, Technology, Scientific Research, and Information Communication Technologies (e.g ISTR). From the interviews it was apparent that the government has continued to fund R&D institutes and universities without paying much attention to the quality of research being conducted, the use of research outputs, or the relationship of research to the country’s economic, scientific, or market priorities. Neither does it appear that efforts have been made to stimulate linkages between the private sector and the universities and research institutes in Rwanda. Most scientists interviewed did not seem to know what other research institutes were doing, nor were there institutional mechanisms to outsource their knowledge to the private sector players like venture capitalists.

In order to successfully exploit the potential of science-based health innovation and biotechnology in Rwanda, several recommendations can be made.

*Recommendation 1: Strengthen the skills base:* For a country with limited resources like Rwanda, investment in knowledge arguably is critical to development. Rwanda should promote the training of its scientists in order to develop a scientific pool that can spur health innovation. This can be done through domestic, regional, and overseas institutions. We realize that this will require considerable resources, but one source of skills and training staff is the Rwandan diaspora who can be encouraged through incentives to provide short-term training to scientists in Rwanda. Attaching skilled members of the diaspora to universities in Rwanda will reduce costs of sending students abroad. Because of its strong links with international institutions, online courses could also be used whereby staff from western universities give lectures on scientific areas as well as on entrepreneurship.

Carrying out an audit of the personnel and scientific infrastructure available in Rwanda will help identify areas that need strengthening. To reduce brain drain, it is important that the government provides incentives through reasonable wages for scientists and clear promotion paths as well as facilities for scientists to be able to carry out their work. Institutions should develop IP policies that reward individuals who are innovative.

*Recommendation 2: Encourage the establishment of R & D within private sector firms:* The government can do this by creating a favourable business environment for firms that want to engage in R & D. Firms like Utexrwa which have already indicated willingness to engage in health innovation might be incentivized. This could be through tax incentives on research equipment and raw materials, as well as local “advance market commitments”, whereby the Ministry of Health guarantees purchase of products developed locally as long as they meet internationally accepted standards. Strengthening of intellectual property regimes and increasing awareness of IP by scientists will be helpful. There is also need to support innovative activities by providing funds that scientists and entrepreneurs can access, such as prototype or product development funds in the form of grants or loans.

*Recommendation 3: Establish a focal point for science-based health and biotechnology development:* There is need to coordinate R & D and innovation at the national level. Currently, there is no National Science Council or Commission which could be the body charged with coordinating research in Rwanda. While the government has established the Rwanda Biomedical Centre (RBC) which merges 15 medical institutions in the country, the focus is limited to clinical research. A council would be charged with capturing the innovative components of biotechnology research. This body would direct sectoral policies – e.g developing a national biotechnology policy – and could clearly define the priorities of the government in terms of health innovation development, as well as concrete actionable steps to realize these goals. It could also identify an institution that will be a centre of excellence for biotechnology. This centre could be equipped with individuals possessing the necessary skills to excel in biotechnology research, as well as the necessary scientific infrastructure, and a mandate to establish relationships between firms and research institutions.

*Recommendation 4: Establish R & D infrastructure and platforms through which knowledge flows from the country’s research institutions will flow to firms in the private sector and vice versa:* These could be in the form of physical platforms or virtual networks [27]. The centres will be equipped with state of the art specialized equipment. From the interviews, scientists cited the lack of advanced equipment as a hindrance to R & D and gave examples of equipment like nuclear magnetic resonance (NMR) machines and high-performance liquid chromatography machines (HPLCs) that do not currently exist in Rwanda. Many types of research instruments are very expensive for individual institutes to buy and maintain. Such a centre can be centrally located, and its facilities accessed by scientists from various institutions as required.

The aim of this study was to describe and analyze Rwanda’s science-based health innovation and biotechnology sector. In doing so we also involved the stakeholders who constitute this sector in developing options to harness Rwanda’s assets and begin to overcome barriers.

Since the completion of our study, we have continued to work with the Ministry of Science and Technology and subsequently the Ministry of Education, to address some of the key challenges we identified in our case study. We jointly hosted a national life sciences workshop held in May 2008 in Kigali where we presented our case study results and recommendations, which were discussed with local stakeholders including government officials, the private sector and the research community. Stakeholders agreed unanimously with the results and recommendations, especially the need to increase knowledge flow. They supported the idea of developing a life sciences innovation center [28]. Since the meeting, an initial disbursement of funds to two projects has been done, and discussions are underway on where the initial start-up activities are to be physically located.

From the study, the main conclusion that can be drawn is that Rwanda is a late bloomer in science-based health innovation and biotechnology, mainly because of events preceding and during the 1994 genocide. There is very little innovative activity occurring in the institutions or in the private sector. The number of scientists is still very low, and even those who are present are not generally motivated to carry out innovation.

However, because Rwanda is still at an early stage in developing its institutions, it is well positioned to shape its institutions in line with current challenges in science-based health innovation and biotechnology; its institutions are not bogged down by the bureaucracy and rigidity that characterize many more-established institutions in Africa [29]. Many of the staff in its institutions are young and amenable to change, and could easily embrace innovation if properly motivated. In addition the country enjoys enormous goodwill among the donor community to implement many initiatives that the government proposes. It also has a large pool of skilled people in the diaspora who are willing to contribute towards developing their homeland. Building on these assets and strong political commitment, Rwanda has the potential to make effective use of science-based health innovation.

## Competing interests

None declared.

## Authors' contributions

KS, PAS, ASD contributed to the concept and design of this study and participated in site visits, analyzed the findings, and participated in manuscript development. MH participated in site visits, and participated in manuscript development.
